# Estrogen mediated expression of nucleophosmin 1 in human endometrial carcinoma clinical stages through estrogen receptor-α signaling

**DOI:** 10.1186/s12935-014-0145-1

**Published:** 2014-12-20

**Authors:** Yunxiao Zhou, Jie Shen, Liqun Xia, Yanli Wang

**Affiliations:** Department of Gynecology, The First Affiliated Hospital, College of Medicine, Zhejiang University, Hangzhou, 310003 China; Department of Pathology, The First Affiliated Hospital, College of Medicine, Zhejiang University, Hangzhou, 310003 China

**Keywords:** Endometrial carcinomas, Nucleophosmin 1(NPM1), Estrogen, Estrogen receptor-α(ERα)

## Abstract

**Background:**

Endometrial carcinoma is one of the most common gynecologic malignancies. Estrogen plays a critical role in its pathogenesis, but the underlying mechanism is not clear. Nucleophosmin 1 (NPM1), a multifunctional protein involved in many cellular activities, has been implicated in the tumorigenesis processes. However, the role of NPM1 in endometrial carcinogenesis remains to be elucidated. The present study was aimed to elucidate the role of NPM1 in different clinical stages of human endometrial carcinoma and the underlying mechanism of NPM1 action.

**Methods:**

The distribution and expression of NPM1 in normal endometrium, FIGO stages I to IV endometrial carcinoma tissues was analyzed using immunohistochemistry, RT-qPCR and Western blotting. The association between NPM1 expression and estrogen and estrogen receptor signaling was investigated in primary-cultured FIGO stage I endometrial adenocarcinoma cells.

**Results:**

A strong positive correlation between NPM1 level and the clinical stage and histological grade of endometrial carcinomas was observed. Expression of NPM1 was up-regulated by estrogen in primary-cultured human endometrial adenocarcinoma cells. Furthermore, estrogen increased NPM1 level via estrogen receptor-α (ERα) signaling, nor estrogen receptor-β signaling.

**Conclusions:**

Expression of NPM1 was gradually increased with the increase of clinical stages of endometrial carcinomas. Overexpression of NPM1 may play a role in the effects of estrogen on the malignant progression of endometrioid adenocarcinoma via ERα signaling. These findings may extend our understanding of the oncogenesis of steroid hormone-related cancers and have significance for the diagnosis and treatment of this carcinoma.

## Background

Endometrial carcinomas is one of the three common female genital tract malignancy, ranking fourth among invasive tumors in women worldwide with 287000 new patients and estimated 74000 deaths per year [1]. In recent years, the incidence of endometrial cancer increased year by year. In China, the morbidity of endometrial cancer increases constantly in population, and the age of onset are younger and younger [2,3]. Based on characteristic epidemiology, clinical symptoms and lesions, two different pathogenetic types of endometrial cancer exist: type I related to estrogen stimulation and type II unrelated to estrogen stimulation [4]. Over 80% of endometrial carcinomas are type I, also known as endometrioid adenocarcinomas [4]. So far, there has been much research on the molecular events estrogen involved that contribute to the development and progression of this disease. But further work is still needed to elaborate the potential mechanism of estrogen action.

Nucleophosmin (NPM, also known as B23 [5], numatrin [6] or NO38 [7]), is a nucleolar phosphoprotein found at high levels in the granular regions of the nucleolus [8,9], and it may shuttle in and out of the nucleolus, and between nucleus and cytoplasm [10]. NPM1 is the mostly studied member of the three NPM isoforms. NPM1 has proved to be a multifunctional protein that is involved in various cellular activities, including transport of pre-ribosomal particles and ribosome biogenesis [11], centrosome duplication [12,13], response to stress-stimuli [14,15], regulation of DNA transcription, maintenance of genomic stability and embryonic development [16], which suggests a role for NPM1 in tumorigenesis. Dysregulation of NPM1 has been found in many solid and hematological malignancies. NPM1 is mutated or aberrantly localized in about one-third of patients with acute myeloid leukaemia (AML) [17]. In addition, NPM1 is reported to be overexpressed in solid tumors of diverse histological origins, including astrocytomas [18], as well as colon [19], hepatocellular [20], bladder [21], breast [22], ovarian [23] and prostate [24] carcinomas. The alteration of NPM1 in human cancer (through overexpression or genetic modification) indicates that NPM1 might play a role as both an oncogene and a tumor suppressor, depending on its dosage and level of expression [25].

However, the role of NPM1 in endometrial carcinomas is still not well-known. Recent research has found NPM1’s expression is associated with the presence of estrogen receptor-α (ERα) in Ishikawa and ARK1 endometrial cancer cells [26]. Moreover, studies in human breast cancer also indicate a hormonal contribution to NPM1 expression and localization [27]. However, no studies which related to human endometrial carcinoma clinical stages were reported. In the present study, we investigated NPM1 alteration in different clinical stages of endometrial carcinoma and analyzed the estrogen regulation of NPM1 expression in primary-cultured International Federation of Gynecology and Obstetrics (FIGO) stage I human endometrial adenocarcinoma cells.

## Results

### Expression of NPM1 was increased with the increase of clinical stages of endometrial carcinomas

**Figure 1:**
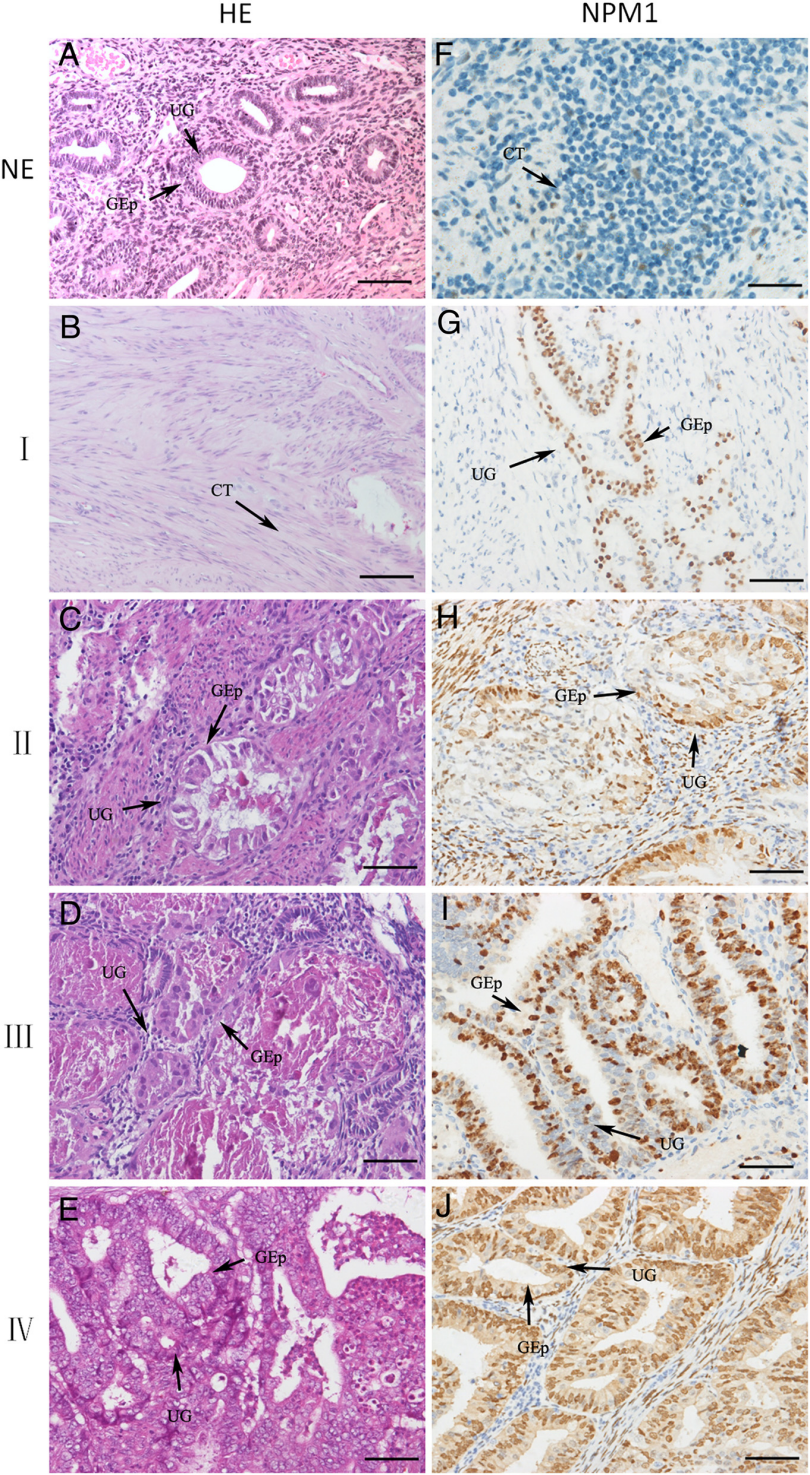
**Immunolocalization of NPM1 in normal endometrium, FIGO stages I to IV endometrial carcinoma tissues.** HE, Morphology of normal endometrium **(A)** and different stages endometrioid cancer tissues **(B, C, D, E)** stained by haematoxylin-eosin. NPM1, NPM1 staining was immunodetected in the glandular cells of all samples. The immunoreactivity of NPM1 was gradually increased from normal endometrium **(F)**, FIGO stages I to IV endometrial carcinoma **(G, H, I, J)**. NE: normal endometrium; I: FIGO stage I endometrial carcinoma; II: FIGO stage II endometrial carcinoma; III: FIGO stage III endometrial carcinoma; IV: FIGO stage IV endometrial carcinoma; UG: uterine gland; GEp: glandular epithelium; Scale bars = 50 μm.

**Figure 2:**
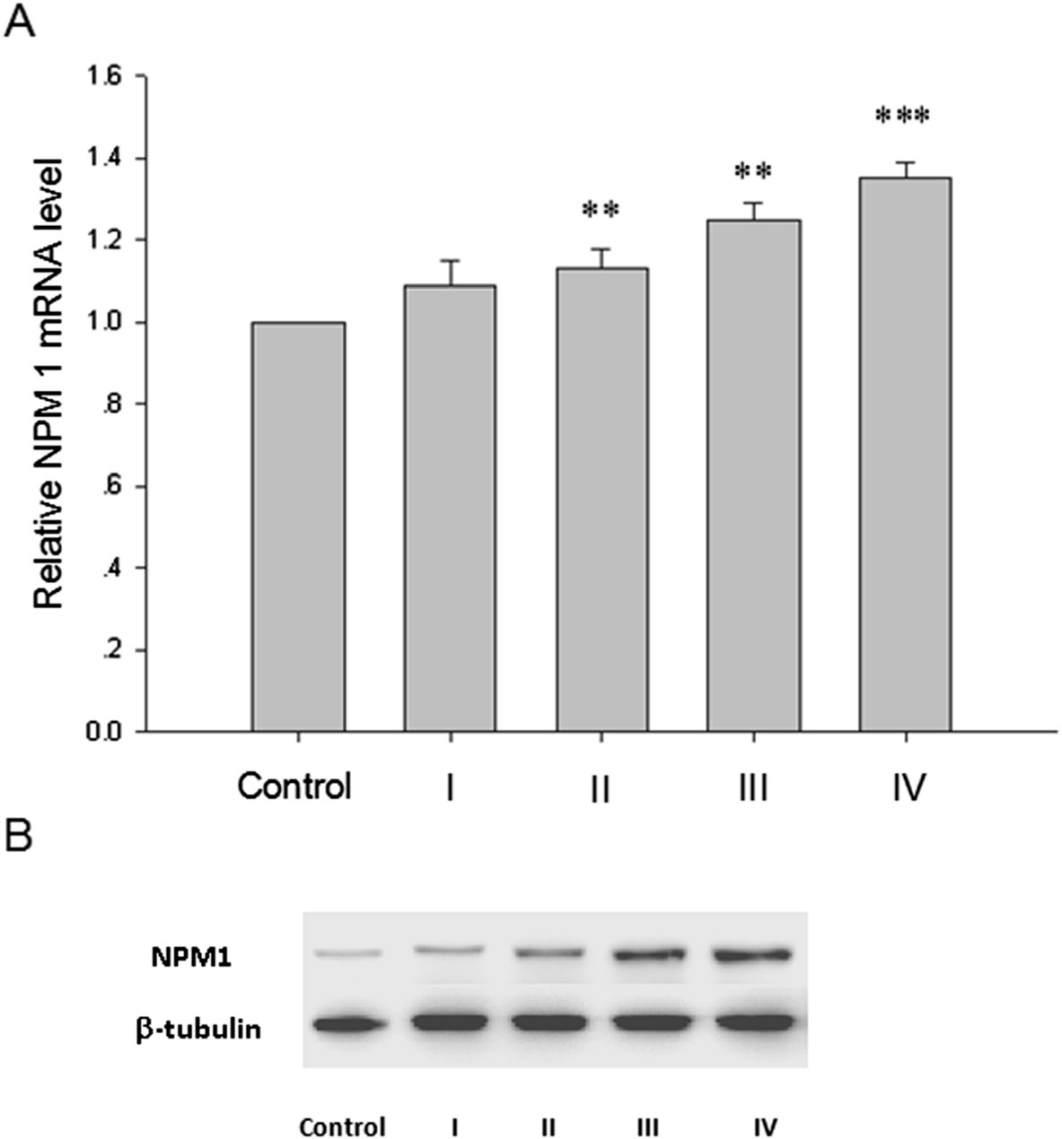
**The relative mRNA level (A) and the protein level (B) of NMP1 were both gradually increased during the carcinogenesis and development of endometrial carcinoma.** Control: normal endometrium; I: FIGO stage I endometrial carcinoma; II: FIGO stage II endometrial carcinoma; III: FIGO stage III endometrial carcinoma; IV: FIGO stage IV endometrial carcinoma; β-tubulin was used as a loading control. *P < 0.05, **P < 0.01, *** P < 0.001 indicate significant differences from the control group.

To determine the distribution of NPM1 in different clinical stages of endometrial carcinoma, NPM1 expression level was investigated in 31 endometrial tissues, including normal endometrium (n = 4), FIGO stage I (n = 8), FIGO stage II (n = 6), FIGO stage III (n = 9) and FIGO stage IV (n = 4) endometrial carcinoma tissues, using IHC, qRT-PCR and Western blotting. NPM1 proteins were stained in the nuclei of glandular cells (Figure 1). Immunostaining intensity of NMP1 gradually increased with the deterioration of endometrial carcinoma (Figure 1), indicating that NMP1 expression is up-regulated in endometrial carcinogenesis and metastasis. Similarly, the NMP1 mRNA level detected by qRT-PCR and the NMP1 protein level detected by Western blotting were also gradually increased with the deterioration of endometrial carcinoma (P < 0.05, Figure 2).

### Expression of NPM1 was up-regulated by estrogen in primary-cultured human endometrial adenocarcinoma cells

**Figure 3:**
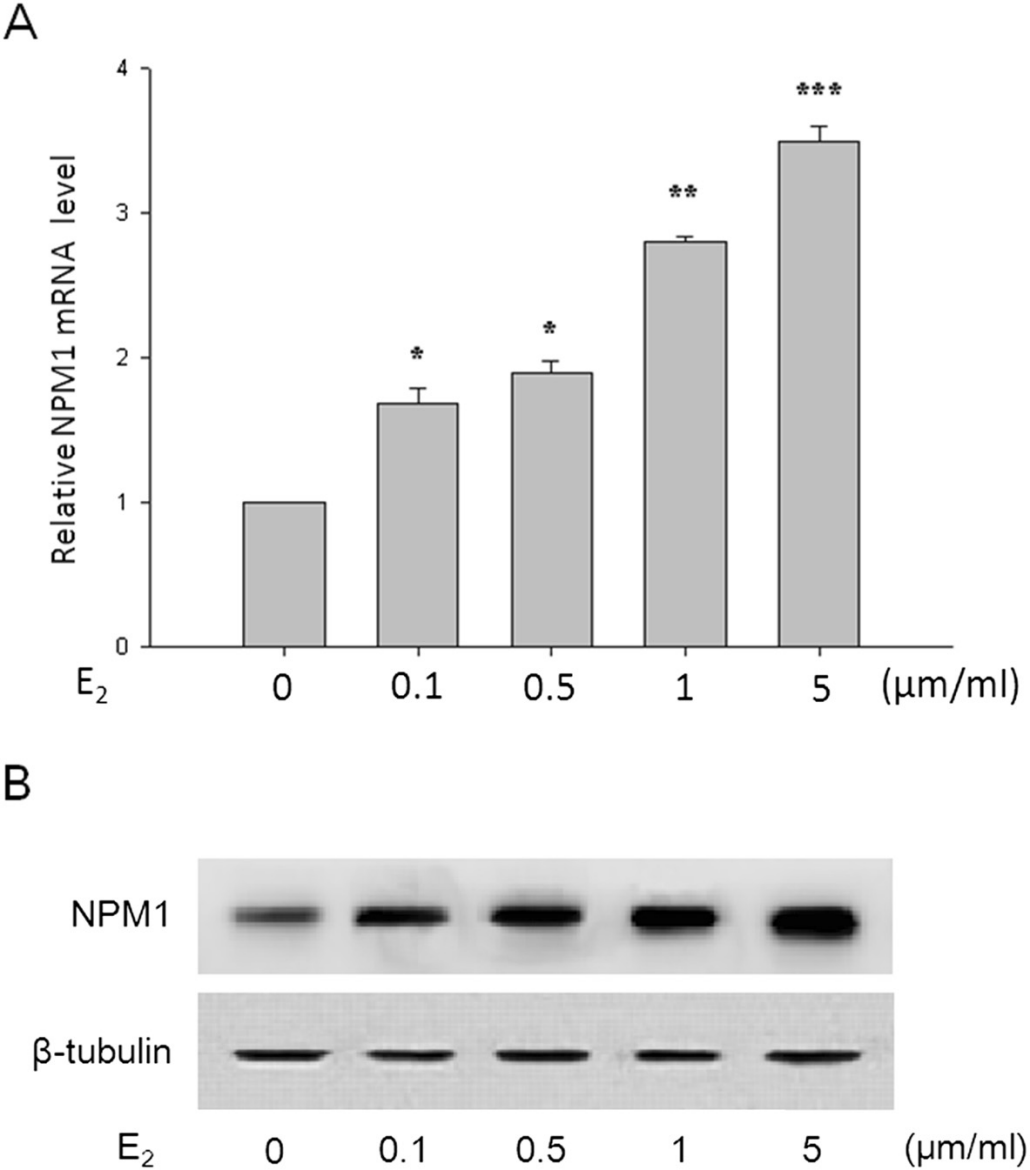
**E**_**2**_ **increased NPM1 mRNA expression (A) and protein expression (B) of primary-cultured FIGO stages I endometrial adenocarcinoma cells in a dose-dependent manner.** E_2_, 17β-estradiol. *P < 0.05, **P < 0.01, *** P < 0.001 indicate significant differences from those treated with 0 μm/ml E_2_.

To investigate if there is a possible hormonal regulation of NPM1 expression, we quantified NPM1 expression levels in primary-cultured FIGO stages I endometrial adenocarcinoma cells stimulated with estrogen (E_2_ 0, 0.1, 0.5, 1, 5 μm/ml) for 24 h using qRT-PCR and Western blotting. As shown in Figure 3, the NPM1 mRNA level of the primary-cultured cells was significantly increased after treatment with E_2_ in a dose-dependent manner, and this result was similar to the protein level detected by Western blotting.

### Expression of NPM1 was regulated through estrogen receptor-α signaling

**Figure 4:**
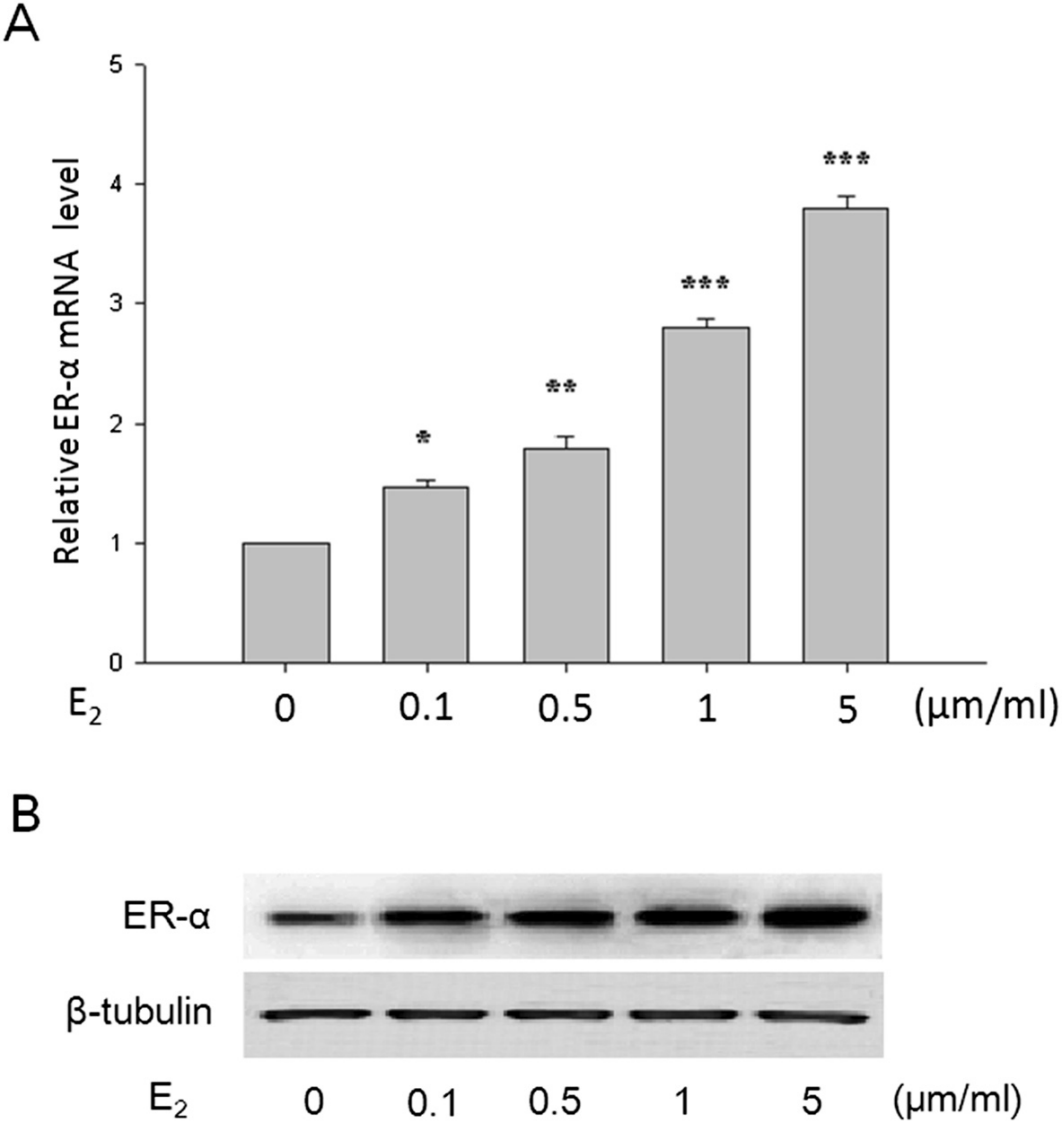
**E**_**2**_ **increased ERα mRNA expression (A) and protein expression (B) of primary-cultured FIGO stages I endometrial adenocarcinoma cells in a dose-dependent manner.** This tendency was similar to that of NPM1 (Figure 3). E_2_, 17β-estradiol. *P < 0.05, **P < 0.01, *** P < 0.001 indicate significant differences from those treated with 0 μm/ml E_2_.

**Figure 5:**
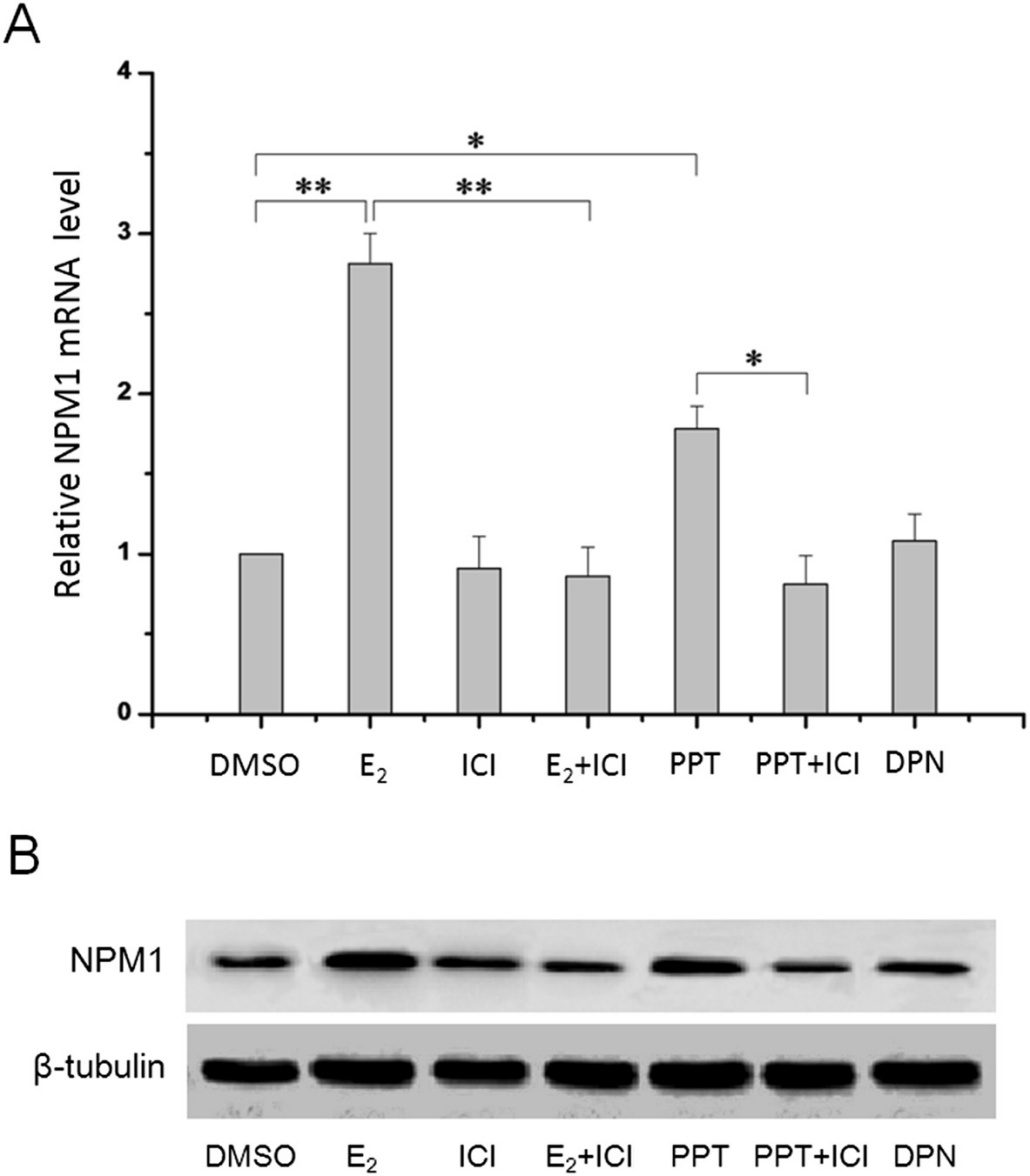
**Expression of NPM1 was regulated via the estrogen receptor-α (ERα) signaling in the primary-cultured FIGO stages I endometrial adenocarcinoma cells.** The NMP1 mRNA expression **(A)** and protein expression **(B)** in primary-cultured cells treated with DMSO, 1 μM E_2,_ 100 nM ICI, 1 μM E_2_ and 100 nM ICI, 1 μM PPT, 1 μM PPT and 100 nM ICI, 1 μM DPN for 24 h, respectively, were detected by qRT-PCR and Western blotting. E_2_, 17β-estradiol; ICI, ICI 182 780; PPT, 4,4′,4″(4-propyl-[1H]-pyrazole-1,3,5-triyl)trisphenol; DPN, 2,3-bis-(4-ydroxyphenyl)–propionitrile. *P < 0.05, **P < 0.01 indicate significant differences from those treated with DMSO.

A previous study demonstrated that E_2_ increased NPM1 protein expression in an ERα-dependent manner in Ishikawa and ARK1 human endometrial cancer cell lines [26]. And the ERα expression as a result of E_2_ treatment in primary-cultured FIGO stages I endometrial adenocarcinoma cells exhibited a similar tendency to that of NPM1 (Figure 4). So we investigated whether the estrogen-regulated NPM1 expression was correlated with estrogen receptor-α in the primary-cultured FIGO stages I human endometrial adenocarcinoma cells. As shown in Figure 5, addition of estrogen receptor-α antagonist ICI 182780 attenuated E_2_-induced mRNA and protein expression of NPM1 in the primary-cultured FIGO stages I endometrial adenocarcinoma cells. In contrast, NPM1 mRNA and protein expression was enhanced upon the stimulation of ERα-specific agonist PPT, while ERβ-specific agonist DPN had no significant effect. ICI 182780 also attenuated the NPM1 mRNA and protein expression increase induced by PPT.

## Discussion

NMP1 has been identified as a molecule involved in development and progress of many human cancers [25]. The NPM1 protein is frequently overexpressed in solid tumours of diverse histological origin, and it has been regarded as a marker for gastric [28], colon [19], ovarian [23] and prostate [24] carcinomas. However, its effect on endometrial carcinoma has not been well-studied. In this study, NPM1 immunostaining frequency and expression level was found to be linked to the clinical stage of endometrial carcinoma, and in vitro studies using FIGO stages I human endometrial adenocarcinoma cells in primary culture demonstrated that ERα-mediated signaling regulated expression of NPM1.

The results of haematoxylin-eosin staining, as shown in Figure 1, revealed significant changes of cellular morphology during the endometrial carcinoma progression. With the development of tumors, the stromal cells initiated malignancy change of cells at first. Then endometrial epithelial cells gradually turned cancerous. As a result, the organized structure of endometrial tissues was more and more difficult to be distinguished.

Overexpression of NPM1 has been reported in many types of major human solid tumors including colon, liver, stomach, ovary, bladder, breast and prostate carcinomas [25]. And expression of NPM1 is higher in many types of tumor and proliferating cells than in normal resting cells [29,30]. Our study using clinical samples showed that NPM1 is significantly more abundant in endometrial carcinoma tissues than in normal endometrial tissues. And furthermore the level of NPM1 is strongly linked to the initiation and progression of endometrial carcinoma. These findings suggested that the overexpression of NPM1 may play an important role in carcinogenesis and development of endometrial carcinoma. And NPM1 may be used as a new biomarker for judging the progression and prognosis of endometrial carcinoma. The correlation between NPM1 and the stage of tumor progression aslo have been observed in some patients. For example, overexpression of NPM1 mRNA was independently associated with bladder cancer recurrence and progression to more advanced disease stages [21].

Prolonged estrogenic stimulation of the endometrium is believed to occur in carcinomas developing not only in the young but also postmenopausal women [31]. Furthermore, it’s also shown that women with endometrial cancer present higher oestrone and oestradiol concentrations compared with healthy women [32-35]. However, the pathogenesis of this disease is less completely understood and the role of estrogens in the initiation and progression of this disease still requires further consideration. It is well accepted that NPM expression and gene integrity are frequently altered in human cancers and aberrantly expressed NPM contributes to tumorigenesis [25,36]. NPM overexpression leads to increased cell growth and proliferation and inhibition of differentiation and apoptosis [25,36]. Here we showed that expression of NPM1 in primary human endometrial adenocarcinoma cells was up-regulated by estrogen in a dose-dependent manner and estrogen increased NPM1 expression via ERα-mediated signaling not ERβ, which are consistent with others’ study with endometrial cancer cell lines Ishikawa and ARK1 [26]. NPM1 was also required for the E2-induced proliferation of endometrial cancer cells [26]. These results suggested a role of NPM1 in the effects of estrogen on the genesis, progress and prognosis of endometrial carcinoma, which may provide new insights into the molecular mechanism of estrogen in endometrial carcinoma.

## Conclusions

Expression of NPM1 was gradually increased with the increase of clinical stages of endometrial carcinomas. Overexpression of NPM1 may play a role via ERα in the effects of estrogen on the malignant progression of endometrioid adenocarcinoma, which may extend our understanding of the oncogenesis of steroid hormone-related cancers and have significance for the therapy of this carcinoma.

## Materials and methods

### Tissue samples

Normal endometrial tissues were collected from patients (n = 21; mean patient age = 52.2 ± 13.31) undergoing hysterectomies for benign gynecological diseases. Endometrial samples from adenocarcinoma (endometrioid and serous types) were obtained from patients (n = 56; mean patient age = 59.4 ± 19.8) undergoing hysterectomy at the Department of gynecology, The First Affiliated Hospital, College of Medicine, Zhejiang University. None had received pre-operative chemotherapy, radiotherapy or endocrine therapy. Patients were evaluated in accordance with the (FIGO) criteria 2009. The number of patients classified as FIGO stage I, II, III and IV were 37, 6, 9 and 4, respectively. This project was approved by the Medical Ethics Review Board of the First Affiliated Hospital, College of Medicine, Zhejiang University, in accordance with the guidelines for the protection of human subjects. Written informed consent was obtained from each participant/guardian.

### Chemicals

17β-estradiol (E_2_) and DMSO were purchased from Sigma-Aldrich (St. Louis, MO, USA). ICI 182 780, 4′,4″,4‴-(4-propyl-[1H]-pyrazole-1,3,5-triyl)tris-phenol (PPT) and 2,3-bis-(4–ydroxyphenyl)-propionitrile (DPN) were purchased from Tocris Cookson Ltd (Bristol, UK).

### Primary culture of human endometrial epithelial cells

The endometrial epithelial cells was derived from stage I endometrial adenocarcinoma and normal endometrium. The primary culture of human endometrial epithelial cells procedures were performed as previously reported [37,38]. After hysterectomy and under aseptic conditions, about 2–4 cm^3^ of cancerous tissue was immediately removed from the endometrium and placed in separate transport tubes containing Dulbecco’s Modified Eagle Medium: Nutrient Mixture F-12 (DMEM/F12) culture medium (Gibco, USA) with penicillin (100 U/ml) and streptomycin (100 mm/ml) for transport to the laboratory on wet ice. The tissues were trimmed of necrosis and washed three in sterile phosphate buffered saline (PBS). Rinsed tissues were diced to 1 mm^3^ pieces, then digested in calcium and magnesium free Hank’s Balanced Salt Solution (HBSS) (Invitrogen, USA) with 2.5 mg/ml collagenase type IV-S (Gibco, USA) at 37°C for up to 1.5 h with gentle agitation. Cells were washed three with 50 ml sterile HBSS and pelleted by centrifugation at 800 rpm at 4°C for 5 min. Washed cells were filtered through a 38 μm autoclaved stainless steel micropore sieves (Newark Wire Cloth Co., USA) to isolate stromal cells and the remaining clumps (containing epithelial cells) washed in HBSS. The clumps were subjected to a second digestion for 30 min before being filtered through 250 μm sieves and 38 μm sieves, respectively. Cells were recovered by inverting and washing the 38 μm sieves and incubated in HBSS with 0.05% trypsin (Gibco, USA), 0.02%EDTA (Sigma, USA) and 0.05 mg/ml DNAase I (Sigma, USA) for 8 min at 37°C. The solution was centrifuged at 1000 rpm at 4°C for 5 min, and washed three in HBSS. Then the cells were resuspended in DMEM/F12 with 10% FBS (Invitrogen, USA), 2 mM L-glutamine (Invitrogen, USA), 100 U/ml penicillin and 100 mm/ml streptomycin and plated in 25 cm^2^ flasks after being assessed for cell viability using trypan blue (Sigma, USA). After 24 hours of incubation, cell cultures were washed twice with PBS, and fresh medium was added. Cells were then passaged using trypsin once the monolayer reached 80–90% confluence and used to seed in new 25 cm^2^ flasks or undergo processing for RNA and protein isolation. By the third passage, few fibroblast and stromal cells were observed. 24 h prior to estradiol treatment, cells were washed in PBS and then grown in steroid-depleted medium lacking phenol red (Invitrogen/Life Technologies, Carlsbad, CA, USA).

### Immunohistochemistry

Representative blocks of formalin-fixed, paraffin-embedded endometrial tissues were cut at 4 μm thick, placed on slides coated with poly-L-lysine, deparaffinized in xylene and rehydrated with a graded series of ethanol. All samples were stained with hematoxylin and eosin for histopathologic examination to select the representative regions. Then after antigen retrieval, quenching endogenous peroxidase and blocking the nonspecific binding, sections were incubated in the mouse anti-human NPM1 antibody (Santa Cruz Biotechnology, Santa Cruz, CA, USA), at a dilution of 1:50, overnight at 4°C. In negative control sections, the primary antibody was replaced with non-immune mouse serum. After washing, the sections were incubated in corresponding secondary antibodies for 2 h at room temperature, followed by incubation with the Strept-Avidin Biotin complex (Dakopatts, Glostrup, Denmark). Peroxidase activity was visualized in chromogen 3,3′-diaminobenzidine solution(Dakopatts, Glostrup, Denmark). After being counterstained lightly with hematoxylin, the sections were dehydrated through an ethanol series to xylene, mounted, examined and photographed. A brown precipitate was considered as positive result.

### Western blotting

Total protein was isolated from the tissue samples or primary-cultured cells using the Tissue or Cell Total Protein Extraction Kit (Sangon, Shanghai, China), respectively, according to the manufacturer’s protocol. The protein concentrations were determined with BCA Protein Assay Kit (Pierce, Rockford, IL, USA). After boiled for 5mins at 100°C, about 30 μg Proteins were separated by 12% SDS–PAGE, and then electro-transferred onto PVDF membranes (0.45 μm, Millipore, Billerica, MA, USA). After blocking with 5% nonfat milk, the membrane was then incubated with the mouse anti-human NPM1 antibody (1:1000; Santa Cruz Biotechnology, Santa Cruz, CA, USA), the rabbit anti-ERα (MC-20) polyclonal antibody (1:1000; Santa Cruz Biotechnology, Santa Cruz, CA, USA), overnight at 4°C, followed by incubation with horseradish peroxidase-conjugated secondary antibody for 2 h at room temperature. The immunoreactivity was then visualized with an enhanced chemiluminescence reaction (Amersham Pharmacia Biotech, Piscataway, NJ, USA) and autoradiography. All the immunoblots were also probed with the rabbit anti-β-tubulin polyclonal antibody (1:1000; Santa Cruz Biotechnology, Santa Cruz, CA, USA) as a loading control.

### Quantitative real-time reverse transcription PCR

Total RNA was extracted from the tissue samples or primary-cultured cells using Trizol reagent (Invitrogen, San Diego, CA, USA), respectively, according to the manufacturer’s instructions. 1 μg of total RNA was reverse transcribed to single-strand cDNA using random exaprimers and 200 U MMLV reverse transcriptase (Invitrogen, San Diego, CA, USA) according to the manufacturer’s directions. Quantitative real-time polymerase chain reaction (qPCR) analyses were performed in a 20 μl reaction volume in triplicate using TransStar SYBR Green qPCR Supermix (TransGen Biotech, Beijing, China), according to the manufacturer’s recommended protocol, and data were collected and analyzed by the ABI Prism 7900 HT instrument (Applied Biosystems, Carlsbad, CA, USA). The transcript levels were normalized to the glyceraldehyde 3-phosphate dehydrogenase (GAPDH) values of the respective sample. Relative quantitative measure of genes was evaluated using 2^-ΔΔCt^ (where Ct is the threshold cycle). The primers for NPM1, ERα and GAPDH were synthesized by Shanghai Sangon Company, Shanghai, China, which are as follows:

- NPM1: forward, 5′-GGGGCTTTGAAATAACACCA-3′;
  reverse, 5′-GAACCTTGCTACCACCTCCA-3′.
- ERα: forward, 5′-AGCACCCTGA AGTCTCTGGA-3′;
  reverse, 5′-GATGTGGGAGAGGATGAGGA-3′.
- GAPDH: forward, 5′-GGTATCGTGGAAGGACTCATGAC-3′;
  reverse, 5′-ATGCCAGTGAGCTTCCCGT-3′.

### Statistical analysis

Data are shown as mean ± standard deviation (SD) for each group. All experiments were performed in triplicate and averaged from >3 independent experiments. Statistical analysis was carried out using SPSS 17.0 software (SPSS, Inc., Chicago, IL, USA). Statistical comparisons between groups were made using one-way analysis of variance (ANOVA) followed by Student’s *t*-test. Value of P < 0.05 was considered statistically significant.

## Abbreviations

AML: Acute myeloid leukaemia
ANOVA: One-way analysis of variance
DMEM/F12: Dulbecco’s modified eagle medium, nutrient mixture F-12
DPN: 2,3-bis-(4–ydroxyphenyl)–propionitrile
E_2_: estrogen
ERα: Estrogen receptor-α
FIGO: International federation of gynecology and obstetrics
GAPDH: Glyceraldehyde 3-phosphate dehydrogenase
HBSS: Hank’s balanced salt solution
NPM1: Nucleophosmin-1
PBS: Phosphate buffered saline
PPT: 4′,4″,4‴-(4-propyl-[1H]-pyrazole-1,3,5-triyl)tris-phenol
qPCR: Quantitative real-time polymerase chain reaction
SD: Standard deviation
